# Enrichment Culture but Not Metagenomic Sequencing Identified a Highly Prevalent Phage Infecting *Lactiplantibacillus plantarum* in Human Feces

**DOI:** 10.1128/spectrum.04340-22

**Published:** 2023-03-30

**Authors:** Xueyang Zhao, Chuqing Sun, Menglu Jin, Jingchao Chen, Lulu Xing, Jin Yan, Hailei Wang, Zhi Liu, Wei-Hua Chen

**Affiliations:** a College of Life Science, Henan Normal University, Xinxiang, Henan, China; b Key Laboratory of Molecular Biophysics of the Ministry of Education, Hubei Key Laboratory of Bioinformatics and Molecular-imaging, Center for Artificial Intelligence Biology, Department of Bioinformatics and Systems Biology, College of Life Science and Technology, Huazhong University of Science and Technology, Wuhan, Hubei, China; c Department of Biotechnology, College of Life Science and Technology, Huazhong University of Science and Technology, Wuhan, China; d Institution of Medical Artificial Intelligence, Binzhou Medical University, Yantai, China; BGI Group

**Keywords:** *Lactiplantibacillus plantarum*, *Lactobacillus plantarum*, bacteriophage, genome annotation, probiotics, gut disease, health

## Abstract

Lactiplantibacillus plantarum (previously known as Lactobacillus plantarum) is increasingly used as a probiotic to treat human diseases, but its phages in the human gut remain unexplored. Here, we report its first gut phage, Gut-P1, which we systematically screened using metagenomic sequencing, virus-like particle (VLP) sequencing, and enrichment culture from 35 fecal samples. Gut-P1 is virulent, belongs to the *Douglaswolinvirus* genus, and is highly prevalent in the gut (~11% prevalence); it has a genome of 79,928 bp consisting of 125 protein coding genes and displaying low sequence similarities to public *L. plantarum* phages. Physiochemical characterization shows that it has a short latent period and adapts to broad ranges of temperatures and pHs. Furthermore, Gut-P1 strongly inhibits the growth of *L. plantarum* strains at a multiplicity of infection (MOI) of 1e−6. Together, these results indicate that Gut-P1 can greatly impede the application of *L. plantarum* in humans. Strikingly, Gut-P1 was identified only in the enrichment culture, not in our metagenomic or VLP sequencing data nor in any public human phage databases, indicating the inefficiency of bulk sequencing in recovering low-abundance but highly prevalent phages and pointing to the unexplored hidden diversity of the human gut virome despite recent large-scale sequencing and bioinformatics efforts.

**IMPORTANCE** As *Lactiplantibacillus plantarum* (previously known as Lactobacillus plantarum) is increasingly used as a probiotic to treat human gut-related diseases, its bacteriophages may pose a certain threat to their further application and should be identified and characterized more often from the human intestine. Here, we isolated and identified the first gut *L. plantarum* phage that is prevalent in a Chinese population. This phage, Gut-P1, is virulent and can strongly inhibit the growth of multiple *L. plantarum* strains at low MOIs. Our results also show that bulk sequencing is inefficient at recovering low-abundance but highly prevalent phages such as Gut-P1, suggesting that the hidden diversity of human enteroviruses has not yet been explored. Our results call for innovative approaches to isolate and identify intestinal phages from the human gut and to rethink our current understanding of the enterovirus, particularly its underestimated diversity and overestimated individual specificity.

## INTRODUCTION

Many lactic acid bacteria (LAB) species have been used in the fermentation processes of foods (1, 2), including fermented dairy, vegetables (3, 4), and meats (5). Some species are recognized as probiotics that can benefit human health (6, 7), inhibit the growth of pathogenic bacteria associated with urinary tract infections (8), and play an important role in the treatment and prevention of intestinal inflammation (9). As a member of the LAB, Lactobacillus plantarum is broadly present in a variety of environments (10, 11). Recently, it was renamed Lactiplantibacillus plantarum (12) and has received a lot of attention as a probiotic powder additive. For example, Ducrotté et al. evaluated the therapeutic efficacy of *L. plantarum* 299v in irritable bowel syndrome and found that after 4 weeks of treatment, abdominal pain in patients and bloating symptoms were relieved compared with those of the control group (13). In addition, Li et al. also studied the effect of *L. plantarum* NCU116 on high-fat-diet-induced nonalcoholic fatty liver disease and found that the bacterium significantly reduced endotoxin and proinflammatory cytokines in rats and regulated colonic flora and hepatic lipid metabolism (14).

Bacteriophages (phages) can infect and replicate within bacteria and thus may pose hurdles to the application of *L. plantarum*. Their infection could disrupt the fermentation of the LAB species, including *L. plantarum*, and lead to slow production and/or substandard fermentation products. In addition, virulent phages can dissolve starter cultures, resulting in low-quality products and economic loss to producers. Due to the industrial importance of the *L. plantarum* strains, it is very important to take effective measures to control phages. In fact, numerous *L. plantarum* phages have been isolated from several sources, including abnormal fermentation liquid (15), anaerobic sewage sludge (16), and organic household waste samples (17). Most of the phages were isolated from locations closely related to food production and have been extensively studied and regularly monitored in factories. Conversely, *L. plantarum* phages have never been isolated from human feces.

In this study, we isolated a novel phage, Gut-P1, from the pooled virus-like particles (VLP) of 35 human fecal samples by using a gut-isolated *L. plantarum* CNGBCC 1800069 strain as the host. In parallel, we also tried to recover the genomes of this phage as well as known *L. plantarum* phages from these 35 samples by using bulk metagenomic next-generation sequencing (mNGS) and VLP sequencing. We analyzed the prevalence of Gut-P1 in the fecal samples and investigated its genomic, morphological, and physiochemical properties. Our results indicated that the Gut-P1 had low sequence similarity to public *L. plantarum* phages and belonged to the *Douglaswolinvirus* genus. The phage was lytic and could strongly inhibit *L. plantarum* at extremely low multiplicities of infection (MOIs). Most importantly, Gut-P1 seemed to be highly prevalent in human feces, which might impede the use of *L. plantarum* as a probiotic in human disease intervention. Strikingly, we did not recover the genome sequence of Gut-P1 from the sequencing data nor identified any significantly similar sequences in public human phageome/virome databases. These results indicate the inefficiency of bulk sequencing in recovering low-abundance but highly prevalent phages and suggest that more efforts are still needed to explore the hidden diversity of the human gut virome despite recent large-scale sequencing and bioinformatics efforts.

## RESULTS AND DISCUSSION

### A novel *Lactiplantibacillus plantarum* phage, Gut-P1, was identified in human feces.

**FIG 1:**
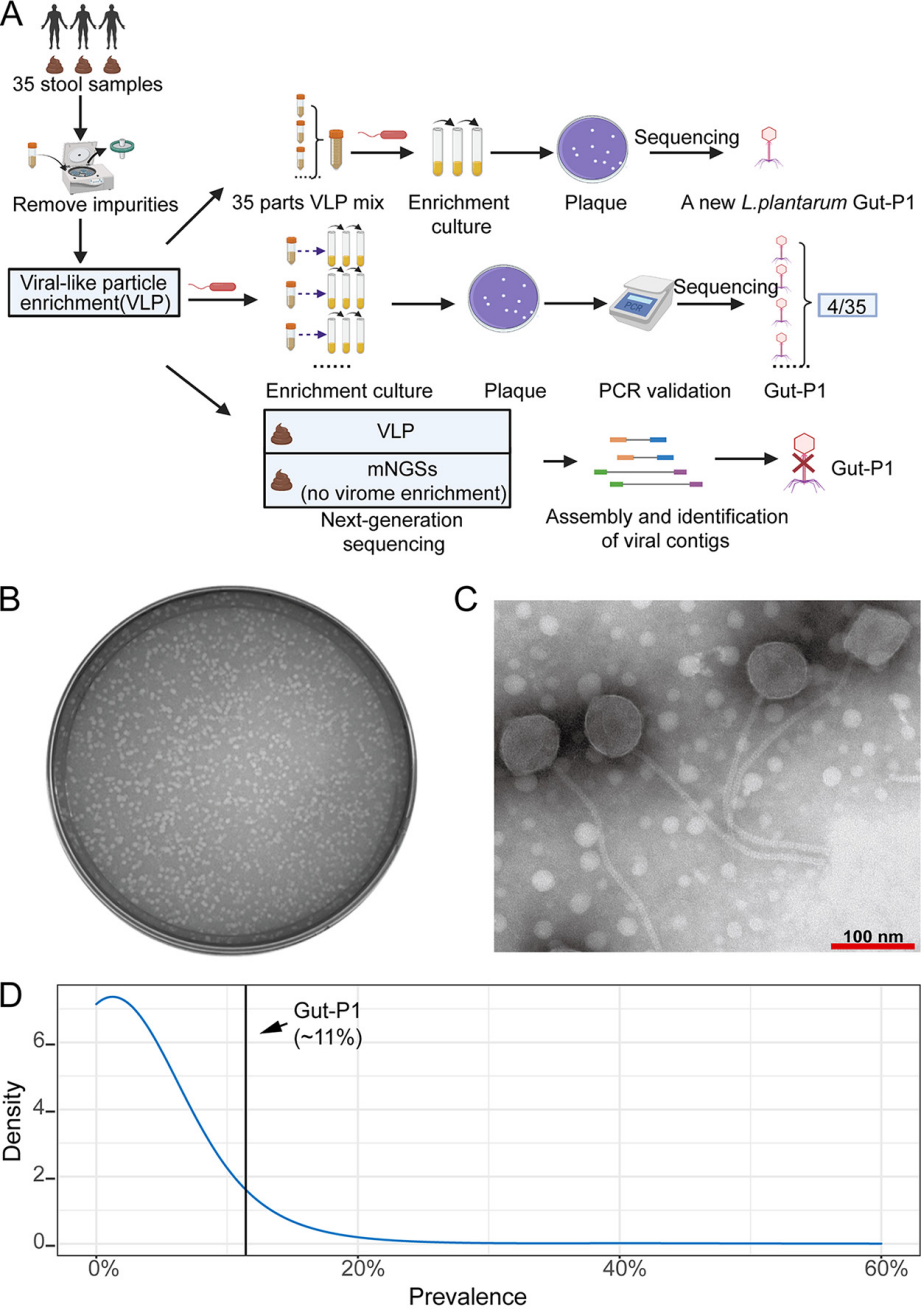
Identification of an *L. plantarum* phage, Gut-P1, that is prevalent in human feces. (A) Systematic screening of *L. plantarum* phages using metagenomic and VLP sequencing and coculture enrichment. (B) Plaques produced on a lawn of *L. plantarum* CNGBCC 1800069 by phage Gut-P1. (C) Transmission electron micrographs of *L. plantarum* phage Gut-P1. (D) Density plot showing the prevalence of the 34,031 viral contigs in the 35 fecal samples according to the vNGS data. The vertical black line indicates the prevalence of Gut-P1 (11%); at this value, Gut-P1 is more prevalent than 93.4% of the 34,031 viral contigs assembled from the vNGS data.

To isolate phages infecting *L. plantarum* in human feces, we incubated a gut-isolated *L. plantarum* CNGBCC 1800069 strain with pooled VLP-enriched supernatants of 35 fecal samples collected from healthy Chinese nationals as volunteers (Fig. 1A) (see Materials and Methods). After three rounds of enrichment (see Fig. S1 in the supplemental material), the supernatant of the final culture was collected and tested for phages by layering it with the host bacterium in MRS broth and agar medium. A phage capable of forming plaques of 2 mm in diameter on *L. plantarum* lawns after 12 h of incubation at 37°C was identified (Fig. 1B). The titer of the phage was 4.68 × 10^9^ phage-forming units (PFU)/mL. We determined the genomic sequence of this phage by next-generation sequencing, followed by bioinformatics analysis (see Materials and Methods). A linear genome of 79,928 bp in length was obtained, and its viral identity was confirmed using the virus prediction tools VirSorter2 (18), VirFinder (19), DeepVirFinder (20), and Seeker (21) (see Materials and Methods). We named it Gut-P1 since it was the first *L. plantarum* phage isolated in human feces.

Electron microscopy analysis showed that Gut-P1 had an isometric capsid of approximately 60 nm in diameter and a long noncontractile tail (approximately 260 nm long and 10 nm wide) (Fig. 1C). These characteristics were consistent with those of other *L. plantarum* phages such as ΦJL-1 according to Lu et al. (22).

**FIG 2:**
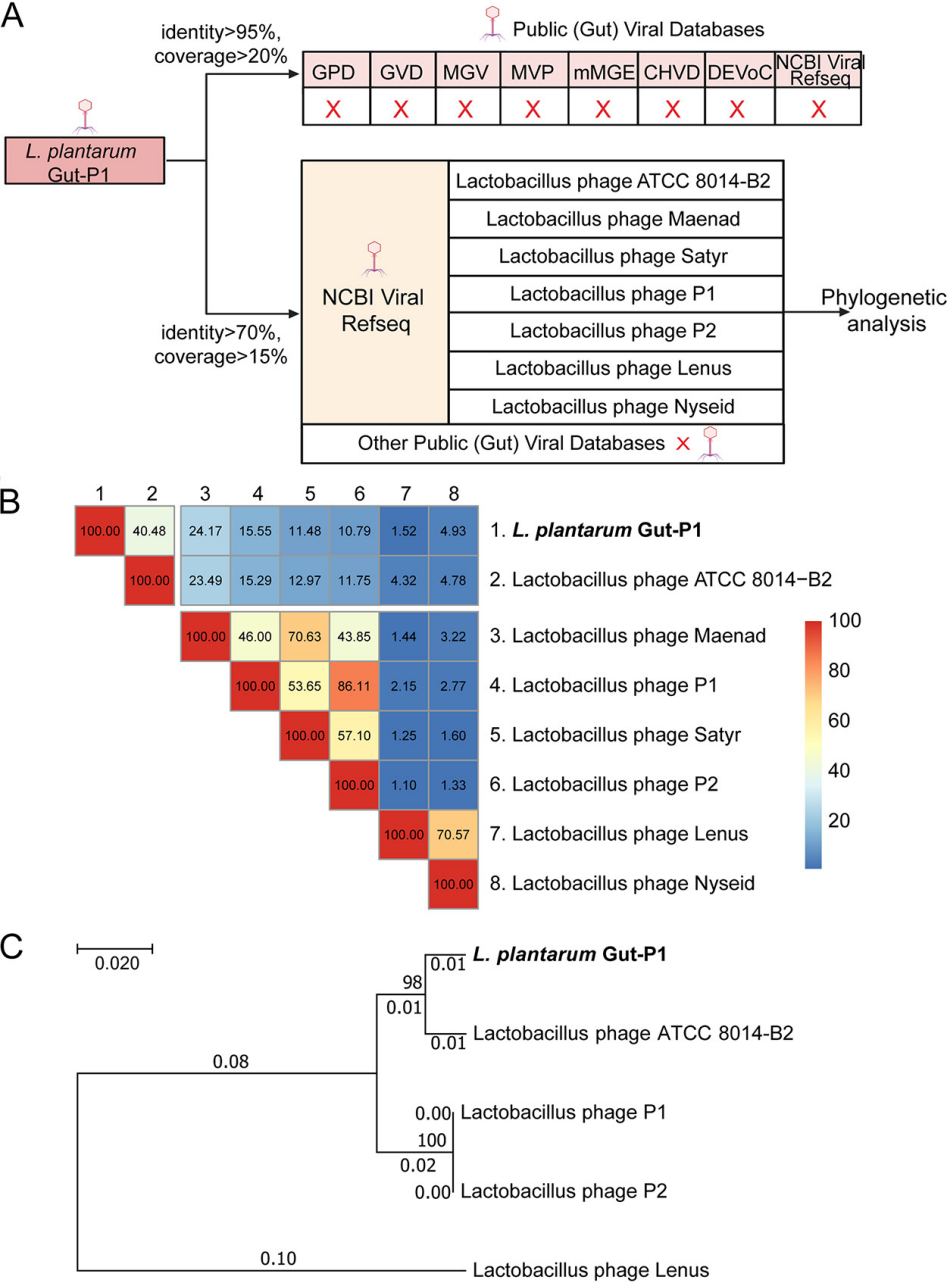
Phylogenetic analysis of Gut-P1 and similar phages in public databases. (A) Search for phages similar to the novel *L. plantarum* phage Gut-P1 by using different BLASTn thresholds in public virome databases, including the NCBI Viral RefSeq (23), GVD (24), GPD (25), MGV (26), CHVD (27), DEVoC (28), MVP (29), and mMGE (30). (B) Heat map plot showing the sequence similarities among the genomes of *L. plantarum* phage Gut-P1 and its seven BLASTn hits in public virome databases. The between-genome scores were calculated using Gegenees, which summarized the overall genome similarities by taking both the BLASTn hit lengths and similarities into consideration; the scores range from 0% (no similarity) to 100% (identical). Blue indicates low similarity, and red indicates high similarity. All other phages used in this figure are *L. plantarum* phages. (C) Phylogenetic analysis of Gut-P1 and its related phages using the large-subunit terminases; phages shown in panel B that did not include the terminase gene were excluded from the analysis.

To check if Gut-P1 was novel, we searched its genomic sequence using BLASTn against viral genomes in the NCBI Viral RefSeq database (23) and recently assembled large-scale human virome databases, including the Gut Virome Database (GVD) (24), Gut Phage Database (GPD) (25), Metagenomic Gut Virus catalog (MGV) (26), Cenote Human Virome Database (CHVD) (27), Danish Enteric Virome Catalog (DEVoC) (28), Microbe Versus Phage database (MVP) (29), and metagenomic Extrachromosomal Mobile Genetic Elements (mMGE) (30). We did not find any BLASTn hit at a rather relaxed threshold of ≥95% of nucleotide identity and ≥20% BLAST coverage of the Gut-P1 genome (Fig. 2A), confirming that Gut-P1 is indeed a novel phage.

### Gut-P1 is highly prevalent in human feces but undetectable by metagenomic or VLP sequencing.

To check the prevalence of the Gut-P1 phage in our samples, we tested each of the 35 VLP-enriched fecal supernatants for *L. plantarum* CNGBCC 1800069 phages by using the same methods as described above. We observed phage plaques on the double-layer plates corresponding to four fecal samples (Fig. S2). Phages from the plaques were then collected, purified, and verified using Gut-P1-specific primers, followed by Sanger sequencing of the PCR products (see Materials and Methods). We confirmed the presence of Gut-P1 in all four fecal samples (Fig. S3), suggesting that this phage could be prevalent in the human gut (i.e., in 4 of 35 samples, corresponding to 11% prevalence) (Fig. 1A). In addition, we also searched the sequence of the PCR product against the phage genomes in the above-mentioned public virome databases and did not find any hits at a threshold of ≥99% nucleotide identity and ≥90% coverage of the query sequence, further confirming the specificity of the PCR results.

Why did the recent large-scale human virome databases fail to recover Gut-P1 if this phage was indeed prevalent in the human gut? These databases often use virus prediction tools such as VirSorter and VirFinder to mine contigs assembled from whole-metagenome (i.e., GPD [25], MGV [26], CHVD [27]) or VLP (i.e., GVD [24] and DEVoC [28]) sequencing data of human (mostly gut) samples. To resolve such an apparent discrepancy, we applied both metagenomic sequencing (mNGS data) and VLP sequencing (vNGS data) to the 35 fecal samples used in this research (see Materials and Methods). We obtained 116,122 and 34,301 nonredundant viral contigs from the mNGS and vNGS data sets, respectively; the contigs were longer than 1.5 kb and classified as viral genomes according to a bioinformatics pipeline adopted from two recent gut virome studies (24, 25) (with modifications; see Materials and Methods). We then ran a BLASTn search against these viral contigs by using Gut-P1 as the query but did not find any significant hits above the threshold of ≥95% nucleotide identity and ≥20% BLAST coverage over the Gut-P1 genome. We further extended our BLASTn search to all contigs assembled from the mNGS and vNGS data sets (without filtering by contig length or the viral recognition pipeline) but still did not find any significant hits. Considering that 4 of the 35 fecal samples did contain Gut-P1, our results indicate the overall inefficiency of metagenomic and VLP sequencing in recovering phage genomes from fecal samples.

The human gut phageome (or virome) is known to be individual specific (31); high-prevalence viruses are often rare. For example, the most abundant and prevalent gut phage clade, crAssphage (32), was found in only 20.6% of the individuals in a Danish population (28). To avoid the noise of low-abundance taxa, genomes with relative abundances lower than 0.01% were considered background noise and removed from the samples. Using this threshold, we estimated a mean prevalence of 2.9% of the 34,301 vNGS data-assembled nonredundant viral contigs across the 35 fecal samples (Fig. 1D; see Table S2 in the supplemental material). We did not use the mNGS data to estimate the viral prevalence, because the current virus prediction pipeline did not distinguish prophages from virions in the mNGS data and thus likely overestimated phage prevalence. Overall, 90.75% of the viral contigs were found in three or fewer fecal samples according to the vNGS data. Thus, with a prevalence of 11%, Gut-P1 could be considered highly prevalent in the human gut, although the PCR- and sequencing-based prevalence values may not be directly comparable.

Together, our findings showed that Gut-P1 was prevalently found in 11% of human feces. However, its genome could not be recovered using metagenomic or VLP sequencing methods.

### Phylogenetic relationships of Gut-P1 and related phages.

To find phages related to Gut-P1, we searched the public virome databases (Fig. 2A) with greatly relaxed BLASTn thresholds (i.e., ≥70% nucleotide identity and ≥15% coverage of the query genome with an E value of <1e−10). We identified in total seven significant BLASTn hits from the NCBI Viral RefSeq database, and all are known to be *L. plantarum* phages (Table S3).

Gut-P1 showed a low degree of nucleotide similarity to other phage genomes in public databases (see Materials and Methods) (Table S3); its closest BLASTn (33) hit was *Lactobacillus* phage ATCC 8014-B2 (GenBank accession no. NC_047739.1) with a nucleotide identity of 87% covering 74% of the query genome (Table S3), which was assigned to the *Douglaswolinvirus* genus and isolated from anaerobic sewage sludge (16). We then performed an all-against-all BLASTn comparison among Gut-P1 and the seven *L. plantarum* phages and visualized the results using a Gegenees tool (34). The results confirmed the overall low similarity between the Gut-P1 genome and all other phage genomes (Fig. 2B). We also constructed a phylogenetic tree using the protein sequences of the terminase gene of these genomes, which encodes an enzyme involved in the packaging of phage DNA into capsids and is a phylogenetic marker used in the investigation of several phage groups (35). We did not find the terminase genes in the genomes of *Lactobacillus* phages Maenad, Satyr, and Nyseid, so the remaining five phages were selected for phylogenetic analysis. The phylogenetic tree confirmed the close relationship between Gut-P1 and *Lactobacillus* phage ATCC 8014-B2; they together could form a separate clade on the phylogenetic tree (Fig. 2C). Therefore, based on the results of the phylogenetic tree analysis and after consulting the latest virus taxonomy on the website of the International Committee on Taxonomy of Viruses (https://ictv.global/taxonomy), we found that Gut-P1 could be assigned to the *Douglaswolinvirus* genus.

Together, our results showed that Gut-P1 is only distantly related to other *L. plantarum* phages.

### Genomic characterization of Gut-P1.

To further understand the genomic differences between the newly isolated *L. plantarum* phage Gut-P1 from the gut and phages isolated from other environments, we analyzed its genomic features, annotated its related functional genes, and predicted its proteins. The results show that Gut-P1 has a double-stranded DNA with a GC content of 36.95% (Table S4), which is significantly lower than that of the other *L. plantarum* phages (42.6%) (17). In total, the Gut-P1 genome comprises 125 genes, including 6 tRNA genes, 110 hypothetical or unsorted protein genes, and 10 genes coding for known viral functions, such as infection (3 genes), packaging (3 genes), replication (3 genes), and assembly (1 gene) (Table S4; annotated using methods described in reference 36). Most of these genes were located at the sense strand, similar to other *L. plantarum* phages (37). We identified a phage terminase (DEDGFLLK_00119) and a phage portal protein (DEDGFLLK_00118) in Gut-P1 that are crucial in the survival of phages; the former initiates DNA packaging, and the latter forms a channel through which the viral DNA is packaged into the capsid and exits during infection (17).

**FIG 3:**
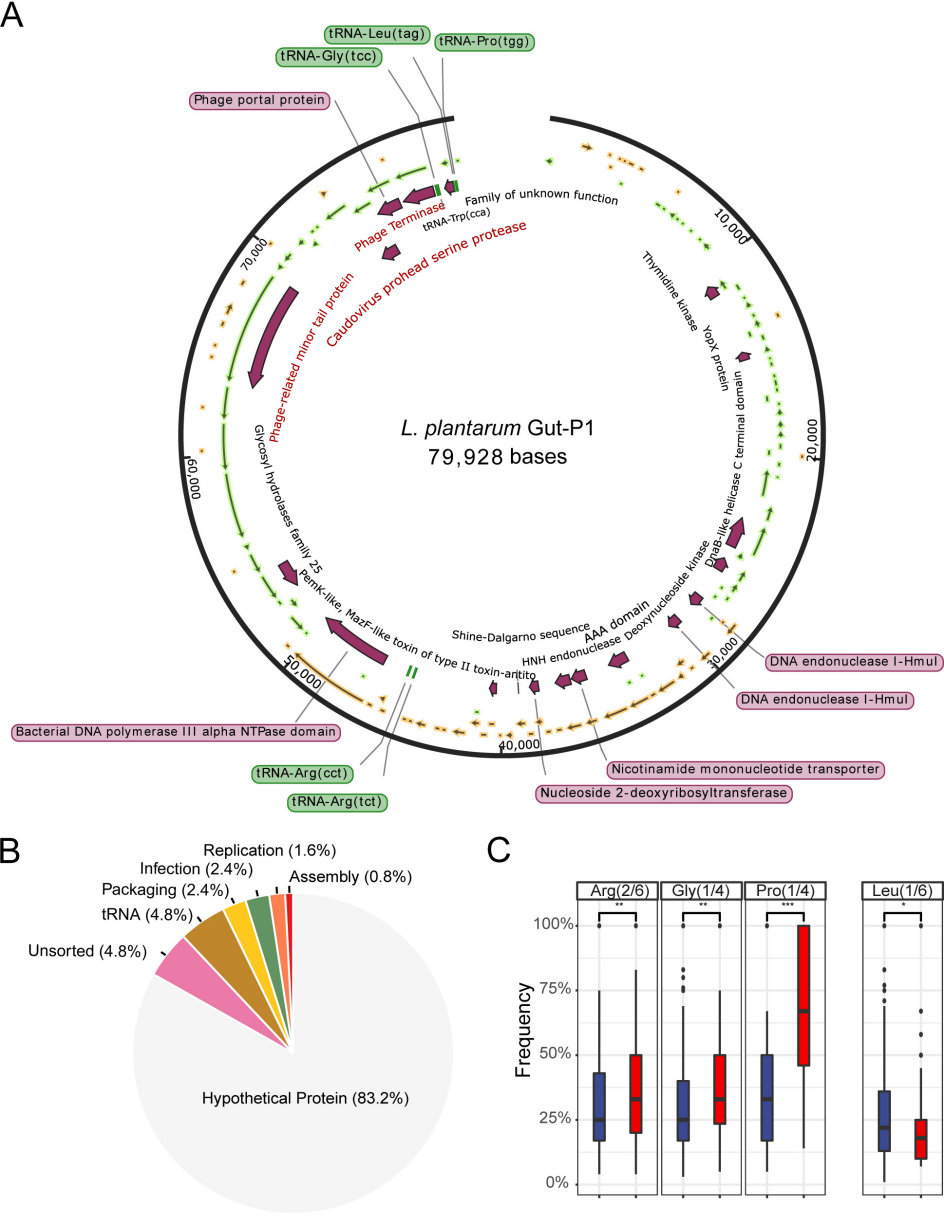
Annotation and characterization of the Gut-P1 genome. (A) Genome map featuring all predicted proteins with assigned functions for phage Gut-P1. (B) Proportions of annotated functional categories of phage Gut-P1 protein coding genes. (C) Usage comparisons of codons corresponding to proteins that are phage encoded (red) or not (blue) in *L. plantarum* phage Gut-P1. Red indicates codons corresponding to phage tRNA genes, while blue indicates codons without phage tRNA genes. Here, we present only amino acids that have multiple codons. The amino acid W (tryptophan) was excluded because it has only one codon (“cca”). The numbers after the amino acid names, e.g., as in Arg(2/6), indicate two out of a total of six codons that code for Arg (arginine) with anticodons corresponding to phage-encoded tRNAs. Wilcoxon rank sum test; *, *P* < 0.05; **, *P* < 0.01; ***, *P* < 0.001.

We also annotated proteins associated with DNA replication, such as the DNA helicase (DEDGFLLK_00053), HNH endonuclease (DEDGFLLK_00072), and DNA polymerase III (DEDGFLLK_00096). The DNA helicase uses the energy derived from the binding and the hydrolysis of (deoxy)nucleoside triphosphates [(d)NTPs] to unwind duplex DNA and RNA structures (38). DNA polymerase III is the primary complex of DNA replication and helps in DNA synthesis (39). The HNH endonuclease is present in many bacteriophages and prophages, highly conserved, and plays a variety of roles in the phage life cycle (40), including packaging, replication, and infection (41). These results were consistent with our annotation that Gut-P1 is a double-stranded DNA phage. We further annotated protein coding genes that were associated with the lifestyle and morphological characteristics of the phage (Fig. 3A). For example, a LysM (42) gene (DEDGFLLK_00084) that codes for a glycosyl hydrolase family 25 protein (43) and a phage tail lysozyme (DEDGFLLK_00108) which has a lysozyme-like fold and the ability to degrade the host cell wall peptidoglycan layer (44) were identified, consistent with the lytic lifestyle of Gut-P1. In addition, we did not identify any lysogen-related genes, further supporting that Gut-P1 is a virulent phage. We also annotated a phage-related minor tail protein, HOQ89_gp113, consistent with the morphological features revealed by electron microscopy (Fig. 1C). In addition, we found six tRNAs (R-tct, R-cct, G-tcc, W-cca, L-tag, and P-tgg) that did not share significant sequence similarities with those encoded in the *L. plantarum* genome, suggesting that Gut-P1 might use its own tRNAs after entering the host cell. These results were also consistent with the previous observation that virulent phages contain more tRNAs than temperate ones (45). Among the six tRNAs, “cca” is the only codon corresponding to the amino acid W (tryptophan), while codons corresponding to three of the remaining four tRNAs were significantly more frequently used by the Gut-P1 protein coding genes than other codons of the same amino acids (*P < *0.01; Wilcoxon rank test) (Fig. 3B and C), indicating that these tRNAs were indeed used for more efficient translation of the phage-encoded proteins. Overall, ~83% of the protein coding genes annotated in the Gut-P1 genomes were functionally unknown, calling for more experimental work to characterize them in the future.

### Physiochemical characteristics of Gut-P1.

**FIG 4:**
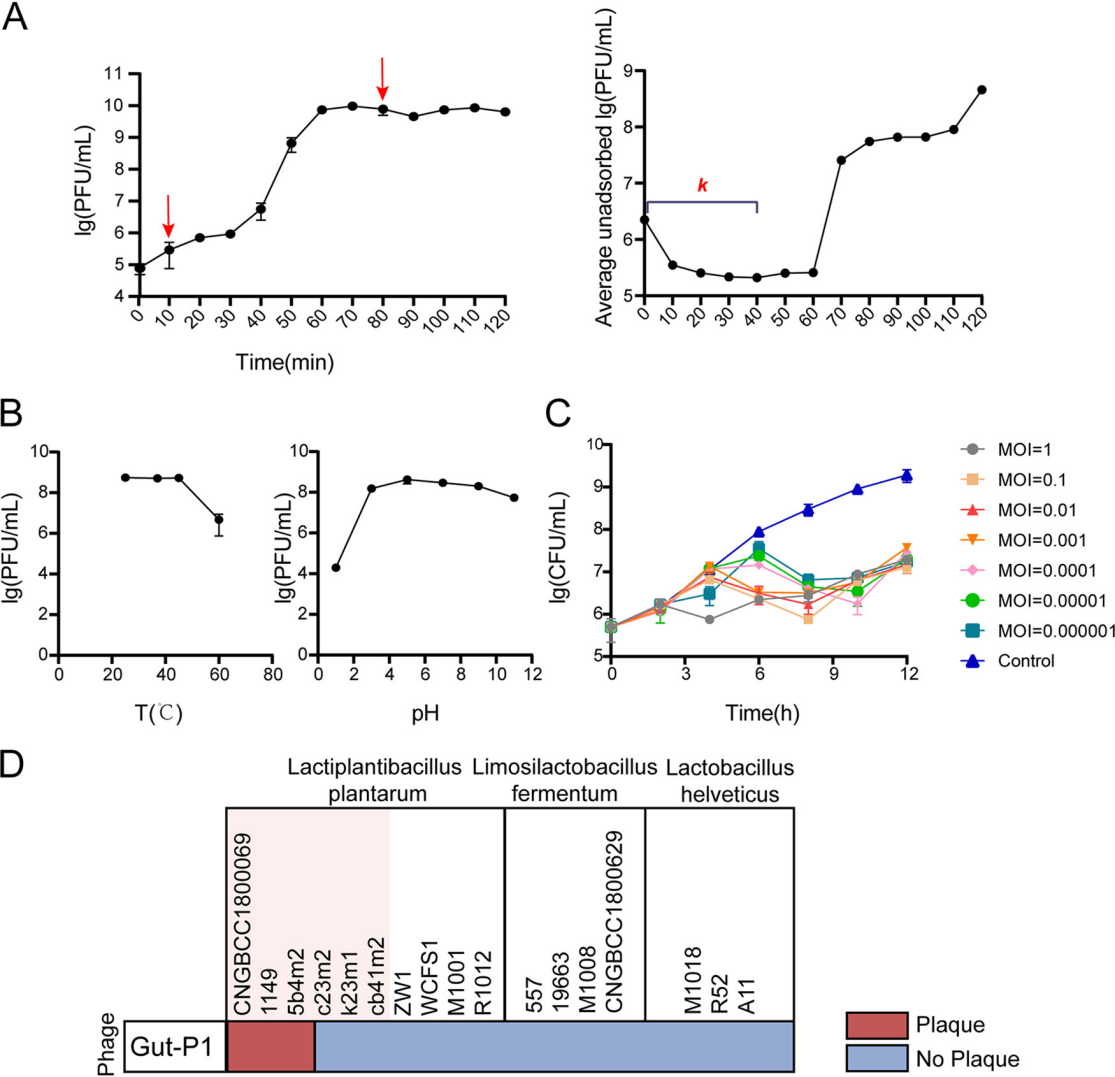
Physiochemical properties and host range analysis of Gut-P1. (A) Left, One-step growth curve of *L. plantarum* phage Gut-P1 and its host *L. plantarum* 1800069 grown in MRS broth. Right, Rate of adsorption of phage Gut-P1 to the host bacterial strain. Data are calculated as the mean ± standard deviation (SD). The experiment was repeated three times. (B) Effect of temperature (left) and pH (right) on phage survivability. For each temperature, phage (500 μL) was placed in SM buffer for 30 min and phage activity was calculated using the double plate method. For each pH, a suspension of phage particles (100 μL) was added to 900 μL of SM buffer at 37°C, and after 2 h, phage activity was calculated using the double plate method. Data are calculated as the mean ± SD of three replicates. (C) Effects of Gut-P1 on the growth of *L. plantarum* CNGBCC 1800069 under different MOIs. Data are calculated as the mean ± SD. The experiment was repeated three times. (D) Host range of phage Gut-P1; the pink background indicates *L. plantarum* strains that are isolated from human intestine/feces.

We next analyzed the physiochemical characteristics of the Gut-P1 phage. The one-step growth curve indicates a burst time of 70 min (Fig. 4A) with a latent period of less than 10 min (Fig. 4A). Gut-P1 showed a shorter latent time than other *Lactobacillus* phages (46, 47). A shorter latent period has been positively related to the lytic activity of the phage, which suggests a stronger growth suppression ability against its hosts (48). The calculated burst size of the phage, the ratio of the total number of the released phage progeny at the end of one growth cycle to the initial count of infected bacterial cells, was approximately 1,270 phage particles per infected cell, which is close to the maximum reported for DNA phages (49). The kinetics of phage adsorption to *L. plantarum* CNGBCC 1800069 cells was determined. After the phage and the host bacteria were cocultured for 10 min, the titer of unadsorbed phages in the supernatant changed from the original 2.06 × 10^6^ PFU/mL to 3.5 × 10^5^ PFU/mL, indicating that about 83% of the phages were adsorbed to the host bacteria within 10 min (Fig. 4A). As shown in Fig. 4A, we observed that the titer of the unadsorbed bacteriophage Gut-P1 in the supernatant started to increase gradually after 40 min of incubation, especially at 60 min, indicating that the bacterial host cells started to release newly generated phages into the culture. Therefore, we calculated the adsorption efficiency of Gut-P1 using the data from the first 40 min and obtained an adsorption rate constant of 6.58 × 10^−9 ^mL/min by using a previously described method (50). Meanwhile, Gut-P1 was able to tolerate high temperatures up to 45°C, and its viability was decreased by 3 orders of magnitude at 60°C (Fig. 4B). This phenomenon was similar to that of the *L. plantarum* virulent phage P2 (51). In addition, Gut-P1 could adapt to a broad pH range, from 3 to 11 (Fig. 4B). The temperature in the human intestine is about 37°C, and the pH is neutral, indicating that the phage can adapt well to the intestinal environment. Meanwhile, in industrial fermentation, the optimum temperature range is 15 to 25°C and the optimum pH is 5 to 8, which means that once the phage contaminates the fermentation product, it will cause huge economic losses (52). We also evaluated the growth suppression ability of Gut-P1 against its host at different MOIs (ratio of phage to host). As shown in Fig. 4C, the lytic ability of phage Gut-P1 was strong. Even when the MOI was 0.000001, the host’s growth could be inhibited (Fig. 4C).

### Host range analysis of Gut-P1.

To test the host specificity of Gut-P1, we selected an additional nine *L. plantarum* strains and seven strains from two related species, Limosilactobacillus fermentum and Lactobacillus helveticus (Table S5). At the same time, we performed 16S sequencing on the laboratory bacterium *L. plantarum* (strains WCFS1, ZW1, 1149, M1001, R1012, 5b4m2, c23m2, k23m1, and cb41m2) and built a phylogenetic tree using the obtained 16S sequences (Table S5) and those of the CNGBCC 1800069 strain and NCBI reference strain NBRC 15891. We used a Lactiplantibacillus songbeiensis strain as an outgroup. As shown in Fig. S4, all the gut strains are closely related to CNGBCC 1800069 and the NCBI reference strains (Fig. S4). We found that Gut-P1 could infect three of six *L. plantarum* strains of intestinal origin, including *L. plantarum* strains CNGBCC 1800069, 1149, and 5b4m2 (Fig. 4D), indicating that Gut-P1 could infect multiple strains of *L. plantarum* of intestinal origin.

### Conclusion.

*Lactiplantibacillus plantarum* (previously known as Lactobacillus plantarum) has been increasingly used as a probiotic to treat many human diseases, but its phages in the human gut remain unexplored. In this work, we isolated and characterized the first *L. plantarum* phage, Gut-P1, from human feces. This new phage has a lytic lifestyle and showed low nucleotide similarity with existing *L. plantarum* phages. It showed broad temperature and pH adaptability and thus could adapt well to the human gut. It targeted multiple *L. plantarum* strains isolated from the human intestine (feces) and could strongly inhibit the growth of *L. plantarum* at low MOIs (e.g., 1e−6). As *L. plantarum* strains have been increasingly used as probiotics to treat intestinal and metabolic diseases, Gut-P1 in the gut may greatly impede their applications. We suggest that more effort be devoted to the future isolation, characterization, and surveillance of *L. plantarum* phages in the human gut.

Interestingly, Gut-P1 was highly prevalent in our cohort (11% prevalence) but undetectable in all of the large human virome databases generated by bioinformatic mining of human fecal metagenome and VLP sequencing data. To resolve this apparent discrepancy, we also submitted all 35 samples to both metagenome and VLP sequencing, including four samples from which Gut-P1 could be isolated. We failed to reidentify the Gut-P1 genome from the sequencing data by using a similar viral identification bioinformatics pipeline. These results indicate the inefficiency of bulk sequencing in recovering low-abundance phages. However, since phages can function at extremely low abundances (e.g., at an MOI of 1e−6 to their hosts), more efforts and new strategies are needed for their efficient discovery. Our results also suggest that we should revisit our current view of the underestimated prevalence of gut phages and point to an unexplored hidden diversity of the human gut virome despite recent large-scale sequencing and bioinformatics efforts.

## MATERIALS AND METHODS

### Recruitment of healthy volunteers and collection of fecal samples.

Thirty-five healthy Chinese nationals as volunteers were recruited in Wuhan, China. Exclusion criteria included (i) any gastroenterological disorders, (ii) use of antibiotics within 2 months, (iii) use of probiotic supplements, and (iv) female volunteers in their menstrual period. Fecal samples were collected from November to December 2020; ~400 to 500 g of feces was taken from each volunteer. The fecal samples were temporarily stored on dry ice, transported to the laboratory, and immediately stored at −80°C.

This study was approved by the Ethics Committee of the Tongji Medical College of Huazhong University of Science and Technology (no. S1241).

### VLP enrichment from human fecal samples.

VLP were enriched from the 35 fecal samples by using a virome enrichment protocol adapted from reference 53 with modifications. Briefly, ~400 to 500 g of frozen feces taken from the −80°C freezer was added with 5 liters of SM (200 mM NaCl, 10 mM MgSO_4_, 50 mM Tris-HCl [pH 7.5]) buffer and stirred by an automated stirrer (A200plus; OuHor, Shanghai, China) at low speed (120 rpm) at room temperature until all feces were dispersed. Then, the suspended mixture was filtered with four layers of gauze (21sx32s/28 × 28) and centrifuged at 5,000 × *g* for 45 min at 4°C. The supernatant was transferred to fresh tubes and centrifuged at 8,000 × *g* for 45 min at 4°C. The supernatant was subsequently concentrated to ~300 mL using a 100-kDa ultrafiltration membrane (Sartorius; Vivaflow 200). The VLP-enriched supernatants were divided into several parts for subsequent experiments and stored either at 4°C for immediate use or at −80°C for storage.

### Isolation, purification, and DNA sequencing of *Lactiplantibacillus plantarum* phages from human fecal samples.

A *Lactiplantibacillus plantarum* (previously known as Lactobacillus plantarum) CNGBCC 1800069 strain isolated from the intestine was obtained from the China National GeneBank (https://db.cngb.org/brc/microbe/CNGBCC1800069) and was used as a host for phage isolation. Briefly, the VLP-enriched supernatants from the 35 fecal samples were mixed. One milliliter of the mix was then added to 9 mL of *L. plantarum* CNGBCC 1800069 culture in early log phase at 37°C for 24 h for phage enrichment. MRS medium (54) was used for *L*. *plantarum* and other bacterial strains used in this study. Supernatants of the coculture were collected and added again with the *L. plantarum* CNGBCC 1800069 culture; this step was repeated twice to further enrich *L. plantarum* phages and eliminate contaminants.

After incubation, the mixture was centrifuged at 8,000 × *g* for 10 min and filtered through a 0.22-μm syringe filter. Plaque and spot assays were then performed in a standard manner using 8 mL of MRS semisolid 0.75% agar powder (Solarbio) as previously described (55, 56), with modifications. Briefly, 100 μL of *L. plantarum* CNGBCC 1800069 culture grown to early log phase in MRS medium and 100 μL of phage sample were added to molten overlay agar tubes kept at 45°C and then vortexed and poured on premade MRS plates. The plates were incubated at 37°C for 24 h. Phages were purified by picking a single plaque, suspending the plaque in 0.5 mL of SM buffer, and subjecting the suspension to a second bilayer plating with the host bacteria. The purification step was repeated three times to obtain plaques of uniform size and morphology before plaque counts.

Next, we amplified and cultured the purified phage in the host strain *L. plantarum* CNGBCC 1800069. After 6 h of incubation, the phage genome was extracted by following a standard phenol-chloroform protocol (57, 58). Briefly, cultures were centrifuged twice at 8,000 × *g* for 15 min at 4°C and filtered with a 0.22-μm filter membrane. The filtrated phage sample was treated with DNase I (1 μg/mL) and RNase A (1 μg/mL) for 30 min at room temperature to digest the exogenous DNA and RNA. Then, the virus was concentrated with NaCl (1 mol/L) and polyethylene glycol (PEG) 8000 (10%, wt/vol) solution overnight at 4°C. Subsequent steps were performed as previously described (59). Briefly, the concentrated crude lysate was treated with chloroform, 8 U of DNase I, 20 U of RNase A, and 3 mM EDTA for 10 min at 65°C. Phage structural proteins were digested for 10 min at 65°C by adding 10% SDS, 20 μL proteinase K, and phage lysis buffer (1/4, wt/vol). Phage DNA was extracted with an equal volume of phenol-chloroform-isoamyl alcohol (25:24:1) and purified using the DNeasy blood and tissue kit. The DNA concentration was detected using a Qubit fluorometer. Sample integrity and purity were detected by agarose gel electrophoresis.

One to 1.5 μg of phage genomic DNA was randomly fragmented by Covaris (S220), and the fragmented DNA was selected by using the Agencourt AMPure XP-medium kit with an average size of 200 to 400 bp. The selected fragments were end repaired, 3′ adenylated, adaptor ligated, and PCR amplified, and the product was recovered by using the AxyPrep Mag PCR cleanup kit. The double-stranded PCR product was heat denatured and circularized by the splint oligonucleotide sequence. Single-stranded circular DNA (ssCir DNA) was formatted into the final library (MGIEasy DNA library preparation kit) and characterized by quality control. The DNBSEQ-T7 sequencer (MGI, Shenzhen) was used for paired-end sequencing with a read length of 150 bp (PE150).

### Assembly and bioinformatic characterization of Gut-P1, a novel *L. plantarum* phage: raw read processing, genome assembly, and identification of Gut-P1.

Raw sequencing reads from the above-described sample were processed by Trimmomatic v0.38 (60) (with parameters LEADING:3 TRAILING:3 SLIDINGWINDOW:15:30 MINLEN:50) to remove adaptors and trim low-quality bases; reads with 50 bp or less after trimming were discarded. Putative human reads were identified from the trimmed samples by aligning the reads to the human reference genome (hg38; GCA_000001405.15) using Bowtie2 (61) (v2.4.2; –end-to-end) and removed from further analysis. The remaining clean reads were assembled using IDBA-UD (62) (release 1.1.3; parameters –maxk 120 –step 10 –min_contig 1000).

In total, two contigs with lengths greater than 10 kb were obtained. The shorter one was 38,590 bp in length and could be aligned to the *L. plantarum* genome with 95.26% nucleotide identity over 75% of the contig length; it was thus excluded from subsequent analysis. The longer one was 79,928 bp in size and did not align to the bacterial genome; it was predicted as viral using VirSorter v2.0 (63) (–min-score 0.7) and VirFinder v1.1 (19) (default parameters). It was thus named *L. plantarum* Gut-Phage 1 or Gut-P1 for short.

### Comparison of Gut-P1 genome with those in public virome databases.

To check if the Gut-P1 genome is novel, we compared its genomic sequence against the NCBI Viral RefSeq database (23) and public human virome databases, including the Gut Virome Database (GVD) (24), Gut Phage Database (GPD) (25), Metagenomic Gut Virus catalog (MGV) (26), Cenote Human Virome Database (CHVD) (27), and Danish Enteric Virome Catalog (DEVoC) (28), and phage genomes in the Microbe Versus Phage (MVP) (29) and metagenomic Extrachromosomal Mobile Genetic Elements (mMGE) databases (30) using BLASTn (33) (v2.12.0+) with default parameters and an E value cutoff of <1e−10.

BLAST hits with ≥95% identity covering 20% of the Gut-P1 genome were considered the same as Gut-P1. These criteria are rather loose; however, none of the viral genomes in existing gut virome databases were identified as Gut-P1. Thus, Gut-P1 was considered a novel phage.

### Experimental validation of Gut-P1 in individual fecal samples using phage plaque formation followed by PCR.

To validate Gut-P1 in individual samples, the 35 VLP-enriched fecal supernatants were cocultured separately with the *L. plantarum* CNGBCC 1800069 for phage enrichment. After three rounds of enrichment as previously described, the supernatants were centrifuged at 8,000 × *g* at 4°C for 10 min and filtered with a 0.22-μm filter. One hundred microliters of the filtered supernatants and 100 μL of the cultured *L. plantarum* CNGBCC 1800069 (OD_600_, ~0.3) bacterial solution were used to spread a double layer for plaque formation. The plaques, if formed, were picked for three rounds of purification. Then, the plaques of the final round were picked, added to 0.1 mL SM buffer, and mixed with a vortexer (Servicebio MX-F). Thirty microliters of lysis solution (TaKaRa; lysis buffer for microorganism to direct PCR) was added. The mixture was then bathed with water at 80°C for 15 min and used as a template for subsequent PCRs using a pair of Gut-P1-specific primers (69F5′-3′, CCTGTACGCTCATTTGCTGA; 69R5′-3′, CTGTATGACCGTGAAGATTACCG). The relevant PCRs are shown in Table S1 in the supplemental material. The PCR products were validated by electrophoresis, purified, and submitted to Sanger sequencing (ABI, 3730XL). The sequencing results were compared with the genome of Gut-P1 using BLASTn (33); to validate the specificity of the PCR, the sequences of the PCR products were also searched against the viral genomes in the above-mentioned public databases using BLASTn.

### Metagenomic and VLP sequencing of the 35 fecal samples.

All 35 fecal samples were submitted to whole metagenomic (no virome enrichment) next-generation sequencing (mNGS). Briefly, total genomic DNA was extracted from 150 mg of feces by using a stool DNA kit (Omega, D4015) according to the manufacturer's instructions. The purified genomic DNAs were quality checked by agarose gel electrophoresis and a Qubit fluorometer (Life Technologies Qubit 2.0). Qualified DNAs were sheared with a g-TUBE (Covaris, USA) to generate a target fragment size of 400 bp. Sequencing libraries were then generated using the MGIEasy universal DNA library prep kit (MGI, Shenzhen, China), according to the manufacturer's instructions, and then sequenced by the DNBSEQ-T7 sequencer (MGI, Shenzhen, China) with paired-end reads of 150 bp. This data set was referred to as mNGS (metagenomic next-generation sequencing) in this study.

Next, 35 stool samples were also subjected to VLP sequencing. Briefly, NaCl was then added to the VLP-enriched fecal supernatants to a final concentration of 0.5 mol/L and stored at 4°C for 1 h, and then PEG 8000 was added to a final concentration of 10% (wt/vol) and then incubated at 4°C overnight. On the following day, phage particles were sedimented at 13,000 × *g* for 35 min at 4°C. The obtained pellets were then fully suspended in ~18 to 36 mL Tris-EDTA (TE) buffer and gently shaken with an equal volume of chloroform. The mixture was centrifuged at 3,500 × *g* for 10 min at 4°C. The aqueous phase was then transferred to a sterile round-bottom flask and evaporated for 15 min using a rotary evaporator at room temperature to remove traces of chloroform which could affect the activity of DNase I in the subsequent step. The aqueous phase was transferred to a new centrifuge tube, and TE buffer was added to recover the volume before treatment with chloroform; DNase buffer was then added to a 1× final concentration. Then, for every 6 mL of supernatant, 50 μL of DNase I mixture (33.3 U/μL; Biolab) and 25 μL of RNase A mixture (0.5 U/μL; Biolab) were added and incubated in a thermostatic oscillator (THZ-C; Peiying, Suzhou, China) at 100 rpm for 30 min at 37°C before the enzymes were inactivated by EDTA buffer (final concentration, 35 mM) and incubated at 70°C for 10 min.

DNA was then extracted from the purified viral particles using a HiPure HP DNA maxi kit (D6322; Magen, Guangzhou, China) according to the manufacturer's instructions. Briefly, proteinase K and SDS lysis buffer were added, and the mixture was then incubated at 56°C for 1 h. Viral particles were further lysed by a CFL buffer provided in the kit, and the lysates were subsequently treated with an equal volume of phenol-chloroform-isoamyl alcohol (25:24:1, pH 8.0), followed by centrifugation at 12,000 × *g* for 15 min at room temperature. After centrifugation, the supernatant was transferred into a new centrifuge tube and treated with an equal volume of chloroform by gentle shaking, followed by centrifugation at 12,000 × *g* for 15 min at room temperature. The aqueous phase was transferred to a new tube, loaded onto a DNA mini column provided in the kit, and centrifuged at 12,000 × *g* for 1 min. The DNA mini column was then washed with buffers GDP and GW2. DNA was eluted using DNA elution buffer and stored at −80°C for further analysis. Note that all buffers and columns used in this part are provided in the kit. The DNA was then quality checked and submitted to library construction and sequencing using the same procedures as described above. The resulting sequencing data were referred as to vNGS (viral next-generation sequencing).

### Sequence assembly and identification of viral contigs from the metagenomics and VLP sequencing data.

Raw sequencing reads from the metagenome (mNGS) and VLP samples (vNGS) were processed by Trimmomatic v0.38 (60) (with parameters LEADING:3 TRAILING:3 SLIDINGWINDOW:15:30 MINLEN:50) for adaptor removal and low-quality base trimming. Human genome reads were mapped (hg38; GCA_000001405.15) using Bowtie2 (61) (v2.4.2; –end-to-end) and removed from further analysis.

The remaining clean reads were assembled using IDBA-UD (62) (release 1.1.3; parameters –maxk 120 –step 10 –min_contig 1000). The mNGS and vNGS data were processed separately. Viral contigs were identified using state-of-the-art viral identification tools, including VirSorter2 (18) (–min-score 0.7) and VirFinder v1.1 (19) (default parameters). Contigs that met one of the following criteria were classified as viral: VirSorter2 score of ≥0.7 or VirFinder score of >0.6, identified by DeepVirFinder (20) or Seeker (21). Assembled sequences were filtered by length greater than 1.5 kb and dereplicated with CD-HIT (64) (v4.8.1; parameters: -c 0.95 -n 8) using a global identity threshold of 95%. In total, 116,122 and 34,031 nonredundant viral contigs with lengths greater than 1.5 kb were identified from the mNGS and vNGS sequencing data, respectively.

### Search for Gut-P1 genome in viral contigs assembled from the mNGS and vNGS data.

To find out if our metagenomics and VLP sequencing efforts could also recover the Gut-P1 genome, the genomic sequence of Gut-P1 was searched against the viral contigs assembled from the mNGS and vNGS data by BLASTn (33) (v2.12.0+) with default parameters and an E value cutoff of <1e−10. BLAST hits with ≥95% identity covering 20% of the Gut-P1 genome were considered the same as Gut-P1. Despite the relaxed criteria, none of the mNGS and vNGS viral contigs were recognized as Gut-P1. Bowtie2 (61) (v2.4.2; –end-to-end) was used in the read recruitment of Gut-P1 in selected vNGS samples.

### Prevalence of 34,301 viral contigs in 35 fecal samples according to the vNGS data.

To estimate the abundance of viral populations, the vNGS clean reads were mapped to the vNGS viral genomes using Bowtie2 (v2.4.2; –end-to-end). Then, we calculated the reads per kilobase per million mapped reads (RPKM) value of each viral genome. Relative species abundance is calculated by dividing the RPKM of a specific viral population by the total RPKM of all viral populations. To avoid the noise of low-abundance taxa, genomes with relative abundances lower than 0.01% were considered background noise and removed from the sample (Table S2).

### Phage morphological characterization by electron microscopy.

Transmission electron microscopy (TEM) was performed as described previously (65). Briefly, purified phage solutions (approximately 10^9^ PFU/mL) were laid onto a carbon-coated copper grid for 5 min and stained with 2% phosphotungstic acid solution for 3 min. Prepared phage samples were viewed using a Hitachi TEM system.

### Phylogenetic analysis of Gut-P1 with other *Lactobacillus* phages.

To find remotely homologous viral sequences to Gut-P1 in NCBI Viral RefSeq (23) and the above-mentioned public viral databases, including GVD (24), GPD (25), MGV (26), CHVD (27), DEVoC (28), MVP (29), and mMGE (30), greatly relaxed BLASTn (33) search criteria (E value < 1e−10, identity > 70%, query coverage > 15%) were used. In total, seven phages were identified only from the NCBI Viral RefSeq database, all of which were *L. plantarum* phages.

The genomic sequences of these phages were downloaded. All-against-all nucleotide alignments were then carried out with Gegenees (34) (v3.1) with default parameters, an alignment heat map was generated based on the sequence similarity (with blue indicating low similarity and red indicating high similarity and the different numbers on either side representing the percentages of this phage in the genome of another phage).

To further reveal the phylogenetic relationships of Gut-P1 with the *L. plantarum* phages, the large terminase protein of Gut-P1 was predicted with Prokka (66) (v1.13; –kingdom Virus), while the large terminase genes from the selected *L. plantarum* phage genomes were extracted according to their annotations in NCBI. The large terminase protein sequences were aligned, and phylogenetic trees were built with MEGA7 (67) with default parameters and then visualized and annotated using iTol (68) and EvolView (69).

### Functional annotation of Gut-P1 proteins.

Gut-P1 genes were predicted using Prokka (66) (v1.13; –kingdom Virus). Proteins translated from the coding DNA sequences (CDS) were annotated with eggNOG mapper v1.0.3-3 (70) and hmmscan (71) v3.3.2 against Pfam (72) v34.0 and VOGdb v204 (E value < 1e−5, score ≥ 50) (http://vogdb.org/).

Phage genes were classified into 11 classes based on the above annotated functions, including LYS (lysis), INT (integration), REP (replication), REG (regulation), PAC (packaging), ASB (assembly), INF (infection), EVA (immune evasion), HYP (hypothetical protein), UNS (unsorted), and tRNA according to a previous study (36).

SnapGene v6.0 (73) was used to visualize the annotation results.

### Codon usage analysis of Gut-P1 protein coding genes.

Six tRNA genes (R-tct, R-cct, G-tcc, W-cca, L-tag, P-tgg) were identified in the Gut-P1 genome by Prokka (66) (v1.13; –kingdom Virus). To find out whether there is a correlation between the existence of the tRNA and the codon preference, codon usage was analyzed with the Codon Usage Calculator (https://www.biologicscorp.com/tools/CodonUsageCalculator). We then extracted those codons that encode the same amino acids as the annotated tRNA anticodon. We found that most codons with a correlated tRNA anticodon are of significantly higher frequency than those that do not (Fig. 3C).

### Physiochemical characteristics and the host range of the newly isolated phage Gut-P1.

The effects of physiochemical parameters on phage infectivity were determined as previously described (51) for Gut-P1, including its one-step growth curve, the effect of pHs and temperatures on phage activity, and the effect of different MOIs on phage infection of the host bacterium. The latent phage period and burst size were inferred from a one-step growth curve as previously described (74), with some modifications. Briefly, an exponentially growing culture of *L. plantarum* CNGBCC 1800069 (1.6 × 10^8^ CFU/mL) was mixed with phage Gut-P1 (6.5 × 10^6^ PFU/mL, MOI of 0.01). To allow for the adsorption of the phage into bacterial cells, the mixture of bacteria and phage was incubated for 20 min at 37°C. After centrifugation at 6,000 × *g* for 10 min at 4°C to remove unadsorbed phage, the pellet was resuspended in MRS broth and then incubated at 37°C with shaking at 200 rpm. Samples were taken every 10 min for 120 min. The titer of bacteriophages was determined by the double-layer plate method. The burst size of phages released per bacterium was calculated from the ratio of the total number of the released phage progeny at the end of one growth cycle to the initial count of infected bacterial cells. Experiments were undertaken independently in duplicate with the duplicate assay.

The assay of adsorption of the phage to *L. plantarum* CNGBCC 1800069 was measured in accordance with a previously reported methodology (74), with some modifications. Gut-P1 (2.06 × 10^6^ PFU/mL) was mixed with *L. plantarum* CNGBCC 1800069 (2.3 × 10^8^ CFU/mL) at an MOI of 0.01, followed by incubation at 37°C without shaking. Samples were taken every 10 min for 120 min postinfection. The samples were immediately centrifuged at 14,000 × *g* for 1 min. The titer of unadsorbed phage was determined by the double-layer plate method. Experiments were undertaken independently in duplicate with the duplicate plaque assay. The adsorption rate constant, *k*, in mL/min, was calculated by using the following equation as previously described (50).

$$k=\frac{2.3}{\mathrm{Bt}}\text{log}\frac{P_{o}}{P}$$

where *k* is the adsorption rate constant (mL/min), *B* is the bacterial cell concentration, *t* is the time, *P_o_* is the starting titer, and *P* is the final titer.

To investigate the effects of temperature on phage activity, 500 μL of phage was placed at 25°C, 37°C, 45°C, and 60°C, respectively, for 30 min. The surviving phage was calculated using the double plate method. In addition, the influence of pHs on the activity of phage was determined at pH 1, 3, 5, 7, 9, and 11. That is, a 100-μL suspension of phage particles was added to 900 μL of SM buffer in which the pH values of the corresponding SM buffer were adjusted by HCl and NaOH. After 2 h of incubation at room temperature, counting was done by the double-layer plate method. Host specificity was also tested using the following species/strains: *L. plantarum* (CNGBCC 1800069, 1149, 5b4m2, c23m2, k23m1, cb41m2, ZW1, WCFS1, M1001, and R1012), *Limosilactobacillus fermentum* (557, 19663, M1008, and CNGBCC 1800629), and Lactobacillus helveticus (M1018, R52, and A11). Among these, six *L. plantarum* strains, CNGBCC 1800069, 1149, 5b4m2, c23M2, k23m1, cb41m2, are of human gut origin.

### 16S sequencing of *L. plantarum* strains and bioinformatics analysis.

To validate the identity of the *L. plantarum* strains collected in our lab, including 1149, 5b4m2, c23m2, k23m1, cb41m2, ZW1, WCFS1, M1001, and R1012, we performed 16S sequencing using the primer pair 341F, 5′-CCTACGGGNGGCWGCAG-3′, and 806R, 5′-GGACTACHVGGGTWTCTAAT-3′, targeting the V3-V4 region of the 16S rRNA gene. The resulting sequences are available in Table S5.

The sequenced 16S reads were processed by Trimmomatic v0.38 (60) (with parameters LEADING:3 TRAILING:3 SLIDINGWINDOW:15:30 MINLEN:50) for adaptor removal and low-quality base trimming. The paired-end reads were then merged using PANDAseq (75) (v2.11; default parameters) before the downstream process. The merged FASTQ was then converted to FASTA format using Seqtk (https://github.com/lh3/seqtk) and dereplicated with CD-HIT (64) (v4.8.1; parameters -c 0.99 -n 8) using a global identity threshold of 99%. The largest cluster’s representative sequence was then selected as its 16S sequence.

For the two representative sequences downloaded from NCBI, i.e., those for *Lactiplantibacillus plantarum* strain NBRC 15891 and *Lactiplantibacillus songbeiensis* strain 398-2, the corresponding regions of the 16S gene were extracted with Seqkit (76) (seqkit amplicon) using the same primers mentioned above, 341F and 806R.

The multisequence alignment of the 16s sequences was created using Muscle (77). Phylogenetic trees were built with FastTree (78) v2.1.10 with default parameters. Phylogenetic trees were then visualized and annotated using iTol (68).

### Data availability.

The assembled and annotated genome of phage Gut-P1 has been uploaded to GenBank under accession number ON117106.

The mNGS and vNGS sequencing data of the 35 fecal samples have been deposited in the NCBI SRA database under accession no. PRJNA947684.

All data generated or analyzed during this study are included in this article (and its supplemental material files).
