# Supplementary material for: Enrichment Culture but Not Metagenomic Sequencing Identified a Highly Prevalent Phage Infecting Lactiplantibacillus plantarum in Human Feces
Source: Microbiol Spectr. 2023 Mar 30;11(3):e04340-22. doi: 10.1128/spectrum.04340-22 (PMC10269749; doi:10.1128/spectrum.04340-22)
Supplement: Supplemental file 6 — Supplemental material. Download spectrum.04340-22-s0006.pdf, PDF file, 5.5 MB [file spectrum.04340-22-s0006.pdf]

Supplementary Figures

Fig. S1. Enrichment culture roadmap

When the host bacterium *Lactiplantibacillus plantarum* CNGBCC 1800069 was cultured in a shaker at 37 °C to an OD_{600} of about 0.3, a mixed fecal filtration was added. After three co-enrichment cultures, the culture medium was centrifuged and filtered, and the obtained supernatant was double-layered with the host bacteria to verify the enrichment of the phages of the host bacteria.

Fig. S2. Phages enriched in 4 stool samples

After enrichment and culture of the host bacterium *Lactiplantibacillus plantarum* CNGBCC 1800069 and four fecal samples, the supernatant after centrifugation filtration was laid in a double layer with the host bacteria at a ratio of 1:1, and incubated at 37 °C for 8h. A.H15; B.H24; C.H7; D.H40.

Fig. S3. Electropherogram of Gut-P1 plaques obtained by enrichment of 35 fecal samples.

The markers in the electrophoresis diagram correspond to the following: Marker 15,000 bp (line M), positive control (line P), negative control (line N), 35 single bacteria, and fecal samples enriched supernatant (lines 1-35). The corresponding primer sequences are 69F5′-3′: CCTGTACGCTCATTTGCTGA, 69R5′-3′: CTGTATGACCGTGAAGATTACCG.

Fig. S4. Phylogenetic analysis of the obtained 16S sequences and those of the CNGBCC 1800069 strain and the NCBI reference strain.

The colored bar aside of the phylogenetic tree corresponds to the following: Other *Lactiplantibacillus* as the out-group downloaded from NCBI (dark blue), the type strain of the *L. plantarum* downloaded from NCBI (yellow), *L. plantarum* strains that are isolated from human intestine/feces (green), host for Gut-P1 (red), non-host for Gut-P1.

Supplementary Tables

Table S1. PCR reaction system.

Table S2. Prevalence of vNGS viral genomes.

Table S3. Information of all available phage records cited by this article.

Table S4. Overall genome statistics of the Lactobacillus phage Gut-P1.

Table S5 Reference information for strains used in the text.

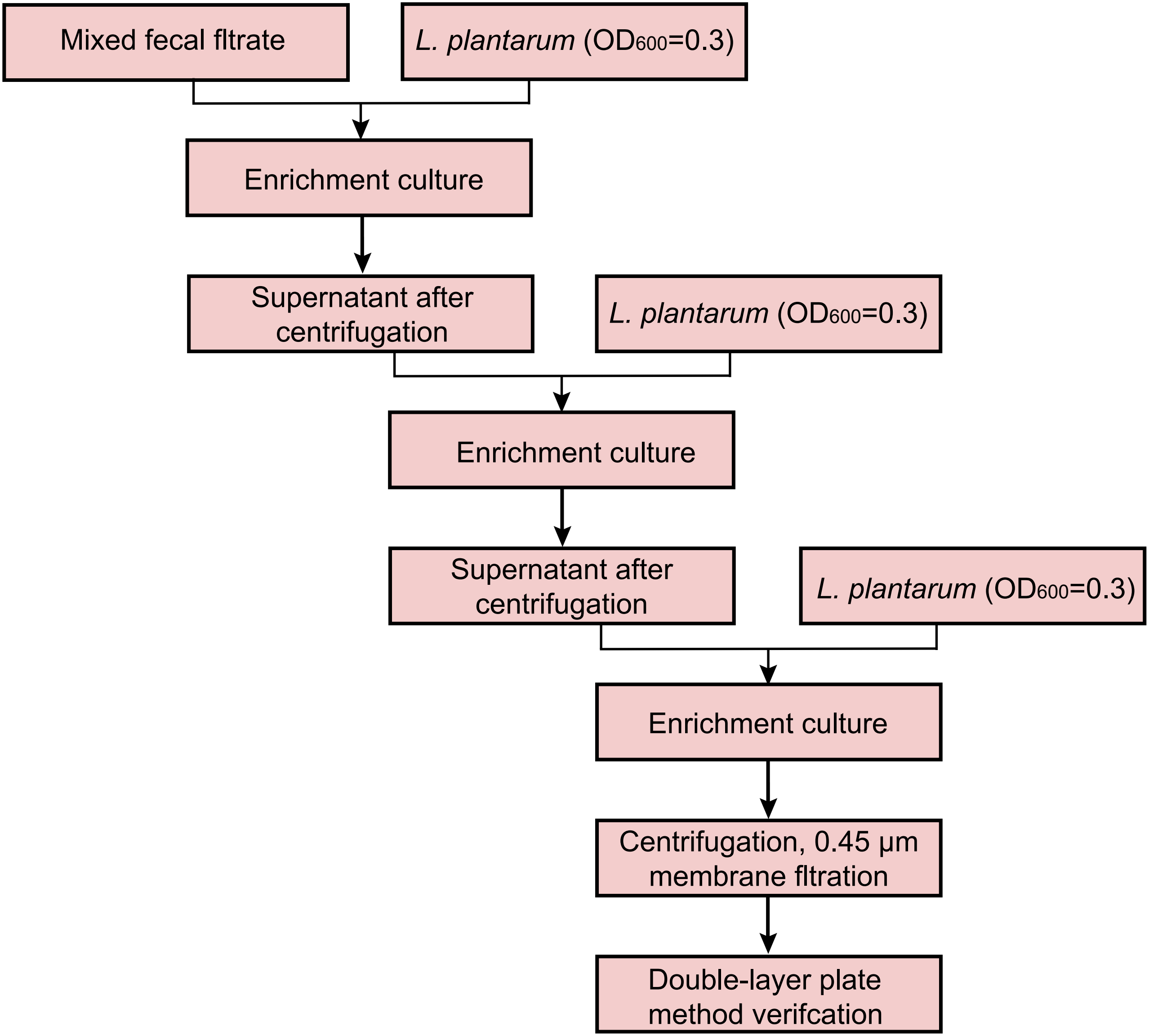
Mixed fecal fltrate
L. plantarum (OD600=0.3)
Enrichment culture
Supernatant after centrifugation
L. plantarum (OD600=0.3)
Enrichment culture
Supernatant after centrifugation
L. plantarum (OD600=0.3)
Enrichment culture
Centrifugation, 0.45 μm membrane fltration
Double-layer plate method verifcation

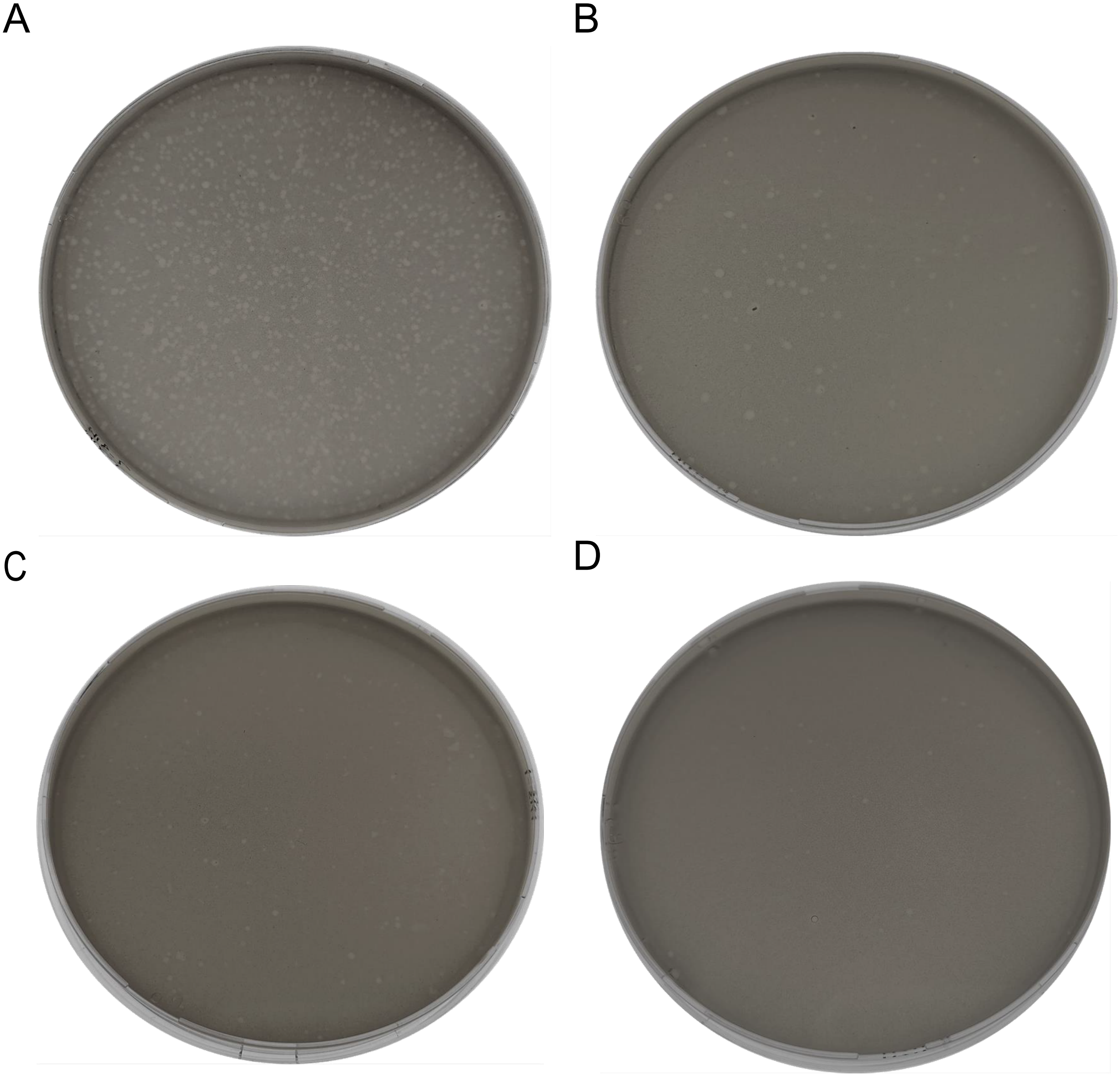
A
B
C
D

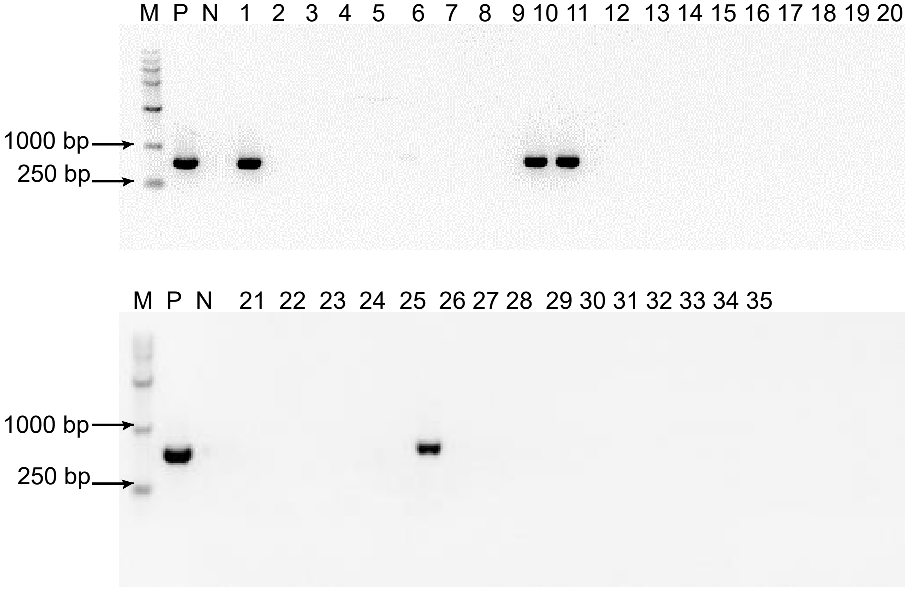
M P N 1 2 3 4 5 6 7 8 9 10 11 12 13 14 15 16 17 18 19 20
1000 bp
250 bp
M P N 21 22 23 24 25 26 27 28 29 30 31 32 33 34 35
1000 bp
250 bp

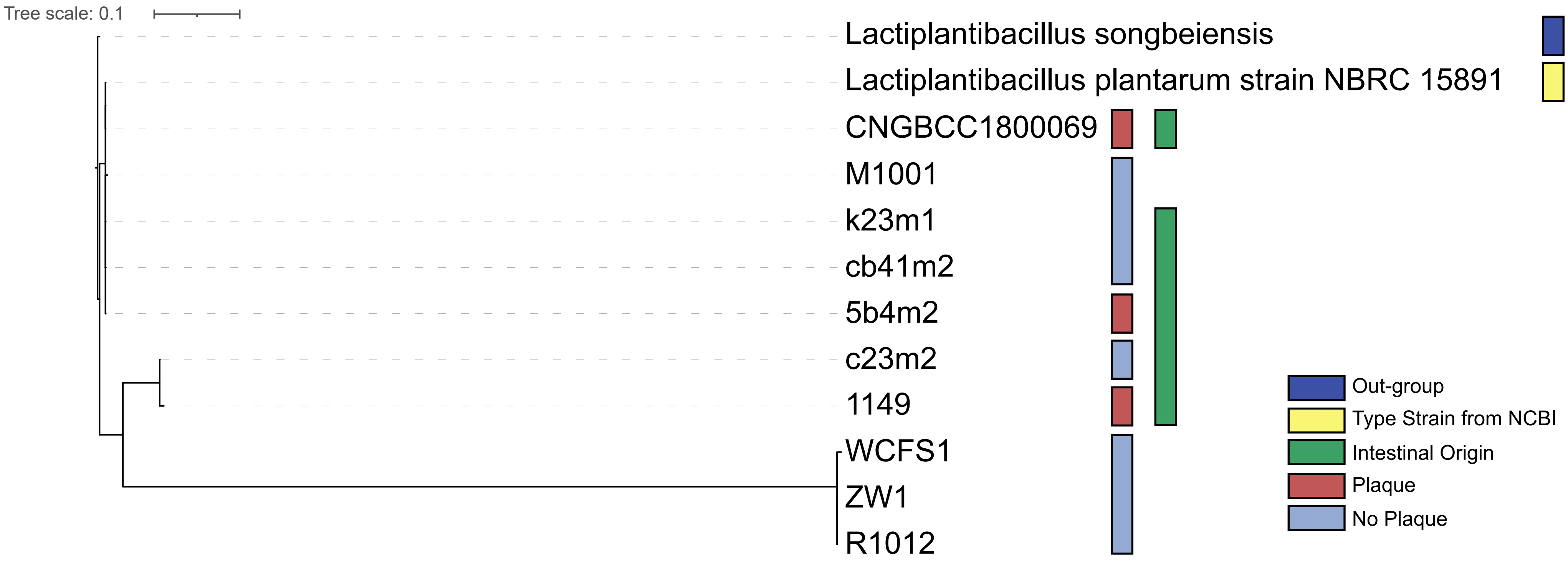
Tree scale: 0.1
Lactiplantibacillus songbeiensis
Lactiplantibacillus plantarum strain NBRC 15891
CNGBCC1800069
M1001
k23m1
cb41m2
5b4m2
c23m2
1149
WCFS1
ZW1
R1012
Out-group
Type Strain from NCBI
Intestinal Origin
Plaque
No Plaque
